# The Many Faces of Child Abuse: How Clinical, Genetic and Epigenetic Correlates Help Us See the Full Picture

**DOI:** 10.3390/children12060797

**Published:** 2025-06-18

**Authors:** Enrico Parano, Vito Pavone, Martino Ruggieri, Iside Castagnola, Giuseppe Ettore, Gaia Fusto, Roberta Rizzo, Piero Pavone

**Affiliations:** 1 Institute for Biomedical Research and Innovation (IRIB), Italian National Research Council (CNR), 95123 Catania (CT), Italy; enrico.parano@irib.cnr.it; 2 Section of Orthopaedics and Traumatology, Department of General Surgery and Medical Surgical Specialties, University Hospital Policlinico “Rodolico-San Marco”, University of Catania, 95123 Catania (CT), Italy; vpavone@unict.it; 3 Unit of Clinical Pediatrics, Department of Clinical and Experimental Medicine, AOU Policlinico “G. Rodolico-San Marco”, University of Catania, 95123 Catania (CT), Italy; m.ruggieri@unict.it; 4 Co.Re.Com, Regional Committe for Communication, 00193 Rome (RM), Italy; icastagnola-cons@regione.lazio.it; 5 Department of Obstetrics and Gynaecology, Azienda di Rilievo Nazionale e di Alta Specializzazione (ARNAS) Garibaldi Nesima, 95124 Catania (CT), Italy; gettore@arnasgaribaldi.it; 6 Department of Biomedical and Biotechnological Sciences (BIOMETEC), University of Catania, 95124 Catania (CT), Italy; gaia.fusto@studium.unict.it (G.F.); rizzo.roberta@studium.unict.it (R.R.)

**Keywords:** child abuse and maltreatment, epigenetics, genetics, hypothalamic-pituitary-adrenal (HPA), post-traumatic stress disorder (PTSD), translational research

## Abstract

Background/Objectives: Child abuse is a pervasive global issue with significant implications for the physical, emotional, and psychological well-being of victims. This review highlights the clinical, molecular, and therapeutic dimensions of child abuse, emphasizing its long-term impact and the need for interdisciplinary approaches. Early exposure to abuse activates the hypothalamic-pituitary-adrenal (HPA) axis, leading to chronic cortisol release and subsequent neuroplastic changes in brain regions such as the hippocampus, amygdala, and prefrontal cortex. These molecular alterations, including epigenetic modifications and inflammatory responses, contribute to the heightened risk of psychiatric disorders and chronic illnesses in survivors. Clinically, child abuse presents with diverse manifestations ranging from physical injuries to psychological and developmental disorders, making timely diagnosis challenging. Methods: A multidisciplinary approach involving thorough clinical evaluation, detailed histories, and collaboration with child protection services is essential for accurate diagnosis and effective intervention. Results: Recent advances in molecular biology have identified biomarkers, such as stress-related hormones and epigenetic changes, which provide novel insights into the physiological impact of abuse and potential targets for therapeutic intervention. Current treatment strategies prioritize the child’s safety, psychological well-being, and prevention of further abuse. Trauma-focused cognitive behavioral therapy and family-centered interventions are pivotal in promoting recovery and resilience. Conclusions: Emerging research focuses on integrating molecular findings with clinical practice, utilizing digital health tools, and leveraging big data to develop predictive models and personalized treatments. Interdisciplinary collaboration remains crucial to translating research into policy and practice, ultimately aiming to mitigate the impact of child abuse and improve outcomes for survivors.

## 1. Introduction

The clinical relevance of child abuse and maltreatment lies in its profound and lasting consequences, which often persist into adulthood. The exposure to early life stress (ELS) implies a general inflammatory response of the whole organism, as an important mediator linking ELS to poor adult health, including developmental delays, mental health disorders such as depression and anxiety, and increased susceptibility to chronic illnesses [1,2,3].

The clinical challenges associated with child abuse are multi-faceted, encompassing not only the immediate need for medical care and psychological support but also the long-term monitoring and prevention of potential sequelae. Healthcare professionals play a crucial role in identifying signs of abuse, providing appropriate interventions, and coordinating with social services to ensure the safety and well-being of the child. However, the complexity of abuse cases, often compounded by family dynamics and societal factors, poses significant difficulties in diagnosis and intervention [1].

Recent advances in molecular research have begun to shed light on the biological underpinnings of child abuse, with studies indicating that early life stress can alter gene expression and immune function [1,2]. These molecular changes may partly explain the increased vulnerability to mental and physical health problems observed in abused children. Our objective was to select literature from the past ten years, focusing on studies or public resources that explicitly adopt or recommend a multidisciplinary approach to the research, diagnosis, and treatment of child abuse victims. Considering that, in recent years, comprehensive reviews have been conducted on various types of child maltreatment—some of which have reassessed victim case histories and pathological outcomes related to digital exposure, and more recently, the impact of the COVID-19 pandemic lockdown—we do not aim to reiterate previous findings [3,4]. Instead, we seek to highlight emerging evidence supporting a more integrated view encompassing key clinical, genetic, and epigenetic components. In this context, the integration of clinical and molecular approaches presents an opportunity to develop more effective strategies for the early detection, prevention, and treatment of child abuse, highlighting the urgent need for interdisciplinary collaboration and continued research in this critical area [1,4].

## 2. Materials and Methods

This literature review was performed according to the guidelines of the Preferred Reporting Items for Systematic Reviews and Meta-Analyses (PRISMA) (http://www.prisma-statement.org/ accessed on 12 January 2025) (Supplementary Figure S1). The period of data collection from PubMed and Google Scholar was selected to span from 2014 to December 2024. To ensure a contemporary perspective, the literature search was limited to these ten years. This timeframe was selected to reflect the most recent advancements in healthcare, including the rise of digital technologies and artificial intelligence, and to emphasize the contrast between technological progress and the persistent gaps in child abuse recognition, treatment, and prevention. The online literature search was organized into two distinct phases to capture both the biological and broader multidisciplinary dimensions of child abuse and maltreatment. The first phase focused specifically on identifying clinical, genetic, and epigenetic evidence—studies that investigated biological markers, physiological responses, or medically relevant outcomes directly associated with abuse-related trauma. The second phase expanded the search to include multidisciplinary correlations, encompassing research that integrated clinical findings with psychological, forensic, social, or educational perspectives, thereby contributing to a more holistic understanding of child abuse.

Inclusion criteria were limited to reviewed studies published in the last ten years (2014–2024), available in English, that provided empirical data with a clear focus on child abuse involving clinical, genetic, or epigenetic components. Studies were required to demonstrate methodological rigor, including appropriate sample sizes and well-defined outcome measures. Exclusion criteria were applied as follows:

Lack of a multidisciplinary component (n = 800): Studies that addressed only psychosocial, legal, or environmental aspects without integrating any clinical, genetic, or epigenetic analysis were excluded, as they did not align with the review’s aim to evaluate an integrated, biologically informed approach.

Insufficient methodological rigor or sample size (n = 769): Studies were excluded if they relied on small sample sizes, lacked control or comparison groups, or were based solely on retrospective self-reports without objective clinical or biological data, as these factors limit the reliability and generalizability of the findings.

The key words used were ‘child abuse’, ‘child maltreatment’, ‘child abuse and maltreatment’, ‘adverse childhood experiences’, ‘early life stress’, ‘early life adversity’, ‘suspected’, ‘violence’, ‘victim‘, ‘maternal/child health’ combined with ‘clinical’, ‘organic’, ‘disease’, ‘illness’, ‘long-term/short-term effects’, ‘diagnosis’, ‘therapy/treatment’, ‘neurological/neuropsychological’, ‘psychological/psychiatric’, ‘behavioral’, ‘gene/genetic’, ‘epigenetic/DNA methylation’, ‘multidisciplinary/interdisciplinary/translational approach’, ‘child protection’, ‘primary care’.

Relevant articles describing the effects of a modern protocol of intervention were manually filtered and analyzed, while duplicates were removed. The primary findings from the search have been incorporated into this reference list.

## 3. Understanding Child Abuse Through the Eyes of Healthcare Professionals

The World Health Organization (WHO) defines child maltreatment as “all forms of physical and emotional ill-treatment, sexual abuse, neglect, and exploitation that results in actual or potential harm to the child’s health, development or dignity” [5,6]. There are four main types of abuse: neglect, physical abuse, psychological abuse, and sexual abuse. Abuse is defined as an act of commission and neglect is defined as an act of omission in the care leading to potential or actual harm [7]. Neglect may include inadequate health care, education, supervision, protection from hazards in the environment, and unmet basic needs such as clothing and food. Neglect is the most common form of child abuse [8]. Physical abuse may include beating, shaking, burning, and biting [9], although the most common signs are bruises. These are often clearly traceable to abusive episodes, especially when located on unusual parts of the body, as discussed later (see NAT signs). The threshold for defining corporal punishment as abuse is unclear [10,11]. Rib, femur, humerus, and tibia fractures are among the most common findings associated with physical abuse [12]. Psychological abuse includes verbal abuse, humiliation, and acts that scare or terrorize a child, which may result in future psychological illness in the child [13]. Sexual abuse is defined as “the involvement of dependent, developmentally immature children and adolescents in sexual activities which they do not fully comprehend, to which they are unable to give consent, or that violate the social taboos of family roles” [14]. Some cases of sexual abuse do not need to involve oral, anal, or vaginal penetration and may include exposure to sexually explicit materials, oral-genital contact, genital-to-genital contact, genital-to-anal contact, and genital fondling [15,16].

Child abuse presents with a multitude of clinical manifestations that can vary widely depending on the type, duration, and severity of abuse. Children subjected to abuse present with a spectrum of physical, psychological, and developmental symptoms, making diagnosis complex. The main warning signs are categorized into: (i) physical indicators, i.e., unexplained injuries, fractures, and burns; (ii) psychological symptoms, i.e., anxiety, depression, posttraumatic stress disorder (PTSD); and (iii) cognitive impairments, i.e., poor academic performance, learning difficulties, and autism spectrum disorders [5,6,7,8]. Sexual abuse constitutes a multifaceted form of maltreatment, encompassing not only physical harm but also profound emotional and psychological impact. Clinical presentations may include trauma to the genital or anal regions, sexually transmitted infections, and age-inappropriate sexual behaviors or knowledge [9,10,11,12].

Alongside these, there are more insidious and subtle symptoms (iii) that may appear later in the course of the growth or in mid-life and can result in a wide range of ELS-related disorders, such as: conduct problems and substance use disorders (SUDs) [13,14,15], metabolic and immune-mediated disorders (i.e., type 1 and 2 diabetes, eating disorders, Crohn syndrome, rheumatoid arthritis) [16,17], cardiovascular disease and elevated blood pressure [18,19,20], respiratory diseases [21,22,23], and resistant epilepsy [24,25,26,27]. Collectively, these (i–iii) may be defined as ELS-related disorders.

Clinicians often face the challenge of identifying these signs (i–iii), as they can be subtle or overlap with those of other medical conditions. Physical abuse may manifest as unexplained bruises, fractures, burns, or lacerations, often in various stages of healing and located in areas not typically injured by accidental means [28,29]. Assessments of suspected non-accidental trauma (NAT) often involve consulting with genetic and metabolic specialists to evaluate patients for rare genetic disorders that may mimic or worsen the signs of child abuse [20,28]. Moreover, the physical abuse of children can involve blunt trauma, thermal injury, and the so-called shaken baby syndrome (SBS) [30]. It can be difficult to draw a clear distinction of child abuse and neglect on the one hand, and acceptable behavior on the other, because of the varying social acceptance of certain child-raising practices.

Clinicians and other healthcare professionals should include caregiver-fabricated illness in the differential diagnosis when confronted with atypical or unexplained medical conditions, even when the caregiver’s behavior appears outwardly exemplary [31]. A significant number of child abuse cases are missed by healthcare providers. For the diagnosis of child abuse to be made, there needs to be a high index of suspicion not only from pediatricians, but also a wider range of physicians. The most common indications for consult have been found to be fractures and subdural hematoma. Consult recommendations for similar indications vary between providers. A standard operating procedure (SOP) with specific recommendations for suspected NAT consults for fractures, intracranial hemorrhage, and other indications was created based on expert reviews and other relevant literature [20,32].

The orthopedist plays a pivotal role in the diagnosis of child abuse, particularly in evaluating fractures and skeletal injuries that may signal NAT [28,32]. Specific fracture patterns, such as posterior rib fractures, metaphyseal corner fractures, and complex skull fractures, are highly suggestive of abuse, especially in infants and young children who are not yet ambulatory. Accurate diagnosis requires not only detailed clinical assessment and imaging but also careful differentiation from underlying medical conditions that predispose to fractures, such as osteogenesis imperfecta (OI), a genetic disorder characterized by bone fragility [28,33]. In such cases, the orthopedist’s ability to distinguish between accidental injuries, abuse, and rare genetic disorders is essential to avoid misdiagnosis. Their expertise is crucial in multidisciplinary teams, contributing to both child protection efforts and the prevention of further harm [33,34].

Dermatologists may not fully conceptualize their crucial role in the evaluation of child abuse and neglect as both mandated reporters and experts in skin pathology [35]. The current information on cutaneous and clinical signs of abuse for dermatologists enables them to gain more insight into the skin examination for child abuse and neglect, develop confidence in their ability to distinguish dermatologic signs of accidental versus inflicted trauma, and more frequently consider abuse and neglect in their differential diagnosis [36]. Given that some cutaneous mimics of child abuse result from a rare disease, they may be more prone to misdiagnosis. For possible child abuse cases in which diagnosis remains uncertain at the time of initial dermatologic evaluation, it is important that dermatologists are prepared to distinguish actual dermatologic conditions in cases of ambiguous skin findings. Additionally, this review will aid clinicians in recognizing the possibility of concurrent actual dermatologic disease and skin findings related to abuse with the acknowledgment that they are not mutually exclusive. A proper recognition of mimics of abuse may prevent unnecessary stress and child protective service investigation [9,36].

Pedodontists play a role in identifying and managing child abuse and neglect within healthcare settings, a crucial responsibility for professionals across various medical disciplines. Notably, pedodontists display a higher level of uncertainty in identifying abuse and neglect cases compared to pediatricians, as demonstrated in a recent increasing number of case reports [37,38,39,40].

Diagnosis of child abuse requires a thorough clinical evaluation, complemented by careful documentation and a multidisciplinary approach. Clinicians should maintain a high index of suspicion and gather detailed histories from both the child and caregivers. This may include observing interactions between the child and caregivers and being vigilant for any inconsistencies in the reported history and physical findings. Radiologic examinations, laboratory tests, and consultations with child protection services may aid in substantiating a diagnosis [29,41]. Timely identification and intervention are crucial not only for the child’s safety and well-being but also for preventing further harm and providing appropriate support and resources for recovery and resilience [1]. Ultimately, it would be valuable to incorporate genetic and epigenetic profiling in cases of child abuse and maltreatment to assess and monitor the molecular signatures associated with ELS-related disorder, as described below.

## 4. Pathophysiological Mechanisms of Child Abuse: A Molecular Perspective

Understanding the clinical manifestations of child abuse is critical for timely diagnosis and intervention, but it represents only one dimension of a broader, more complex picture. Increasing evidence indicates that ELS, such as that caused by abuse and neglect, can lead to long-lasting biological changes. These changes are often mediated through genetic susceptibilities and epigenetic modifications, which influence the individual’s physiological and psychological response to trauma. Therefore, a deeper exploration of the underlying molecular mechanisms is essential to fully grasp how childhood abuse translates into chronic disorders across the lifespan.

Child abuse is a complex issue with profound clinical and molecular implications, as it can induce a cascade of physiological and biochemical changes in the developing brain and body, ultimately affecting both the immediate well-being and long-term health of victims. Understanding the pathophysiological mechanisms from a molecular perspective is essential to grasp its full impact. At the molecular level, stress responses initiated by abuse activate the hypothalamic-pituitary-adrenal (HPA) axis, leading to the chronic release of stress hormones like cortisol [42,43,44]. Persistent elevation of cortisol can produce neuroplastic changes in the brain, potentially affecting regions such as the hippocampus, amygdala, and prefrontal cortex, which are pivotal for emotional regulation, memory, and executive function. Dysregulation of these areas is linked to heightened risks of psychiatric disorders, including anxiety, depression, and PTSD. The latter, when triggered by episodes of child abuse and maltreatment, is also recognized as a distinct entity named ‘child abuse syndrome’ (CAS) [45]. Analogously, eating disorders are distinguished from the eating disorder symptoms associated with different types of child abuse [46]. Genetic susceptibility plays a significant role in shaping an individual’s response to stress, with specific gene variants influencing the regulation of stress-related pathways, such as the HPA axis and neurotransmitter systems [47]. However, genetic predisposition alone does not determine outcomes; adverse environments, such as child abuse and maltreatment, can interact with genetic vulnerabilities to exacerbate the risk of developing psychiatric disorders [48]. Conversely, supportive and nurturing environments may buffer genetic risks, highlighting the dynamic interplay between nature and nurture in determining resilience or susceptibility to stress-related conditions, as reviewed by Breton et al [49].

Additionally, epigenetic modifications, specifically DNA methylation and histone modification, may alter gene expression patterns, further predisposing individuals to behavioral and emotional dysregulations [50]. This molecular dysregulation can impede normal neurodevelopmental trajectories, potentially manifesting in cognitive deficits and social dysfunctions. Moreover, inflammatory pathways might be perpetually activated, leading to an increased susceptibility to various health issues like autoimmune disorders. These molecular insights highlight the critical need for early intervention and treatment strategies to mitigate the long-lasting effects of child abuse on mental and physical health [4,51]. One recognized mechanism is the stress effect on cancer vulnerability, and in particular, shortening telomers paving cell longevity [52,53,54]. Beyond the tumor predisposition, an amount of evidence sustained that ELS is the triggering factor for the genome instability and chromosomal length changes underlying the vulnerability to develop many diseases [55,56,57,58]. However, there are mixed results elucidating that the telomere length in children is a reliable marker of a passed ELS experience, especially in relation to other clinical characteristics, such as the brain morphometry [59,60]. Advancements in neuroimaging have facilitated the visualization of abuse-related structural and functional brain changes. Functional MRI (fMRI) studies have revealed reduced hippocampal volume in individuals with histories of childhood trauma; hyperactivity in the amygdala [61] correlating with increased anxiety and PTSD susceptibility [45]; and connectivity disruptions in the prefrontal cortex, affecting executive functioning [62]—with greater difficulties in children with a history of sexual abuse [63].

In the absence of more structured and numerically larger data, the ability to identify whether or not telomeres are shortened following trauma is not a discriminating tool, but it would be useful to re-evaluate and monitor the impact on replication stress and on accumulation of DNA damage, leading to the risk of developing premature aging diseases.

## 5. Biomarkers and Molecular Correlates in Child Abuse Research

Recent advancements in molecular biology have opened avenues for understanding the complex underpinnings of child abuse through the identification of biomarkers and molecular correlates. Biomarkers, which are biological indicators found in blood, other bodily fluids, or tissues, have the potential to offer unique insights into the physiological and psychological impact of child abuse. Several studies have identified alterations in stress-related hormones such as cortisol, which can be dysregulated due to chronic stress or trauma [64]. Such hormonal imbalances are indicative of the body’s prolonged activation of the stress response system, often seen in children who have experienced abuse [65]. One of most debated research topics is focused on the detection and characterization of genetic and epigenetic changes associated with child abuse to find a new, more informative class of health indicators of ELS-related disorders. Initially, research on the individual response to stressors followed divergent paths: some authors have primarily investigated genetic anomalies, such as risk variants, that predispose individuals to ELS-related disorders, while others have focused on how ELS induces alterations in the methylation patterns of genes influencing mental health outcomes, both in genetically susceptible and non-susceptible individuals. Later, studies converged to focus on the combined analysis of epigenetic changes alongside the presence or absence of risk genotypes according to the increasing importance of gene–environment interaction (GxE) in shaping risk and resilience to ELS disorders [66]. Given that the psychiatric component of ELS-related disorders has been the most extensively investigated, we have summarized studies examining how certain genetic variants may influence this susceptibility [67] (Table 1).

The *FKBP5* (FKBP prolyl isomerase 5) gene plays a role in regulating the stress hormone system. Certain polymorphisms in *FKBP5* have been linked to heightened stress responses and an increased risk of PTSD symptoms in individuals who have experienced childhood trauma [68]. The brain-derived neurotrophic factor (*BDNF*) gene is crucial for brain development and plasticity. The Val66Met polymorphism in *BDNF* has been associated with altered stress responses. Some studies suggest that individuals with the Met allele may have a higher risk of developing depression following childhood maltreatment [69].

The serotonin transporter gene (*SLC6A4*) contains a polymorphism known as the serotonin (5-HTT) transporter length polymorphism (LPR), 5-HTTLPR. The short (s) allele, in contrast with the long (l) version, of this variant has been linked to increased vulnerability to major depressive disorder (MDD) in the context of stressful life events, including childhood abuse [70]. The monoamine oxidase A (*MAOA*) gene is involved in the metabolism of neurotransmitters. A variable nucleotide tandem repeat (VNTR) polymorphism in the *MAOA* promoter region influences transcriptional efficiency, producing high-activity (*MAOA-H*) or low-activity (*MAOA-L*) alleles, with *MAOA-L* linked to increased impulsivity and greater risk of antisocial behavior following childhood maltreatment [71].

The corticotropin-releasing hormone receptor 1 (*CRHR1*) gene influences the body’s response to stress. Some haplotypes of *CRHR1* have been found to confer resilience against depression in individuals with a history of childhood abuse, highlighting the complex interplay between genetics and environmental factors [72].

However, studies have shown that higher polygenic risk scores for ELS-related disorders are associated with an increased likelihood of experiencing childhood abuse, such as the combination of *5-HTTLPR* and *BDNF* polymorphisms [70]. This suggests a GxE correlation where genetic predisposition may influence the risk of encountering adverse experiences, which in turn can exacerbate psychiatric symptoms [67,73]. Therefore, much greater importance has been given to the study of epigenetic modifications in childhood abuse.

Unlike genetic mutations, these changes do not alter the DNA sequence, but instead regulate gene expression, and are highly sensitive to environmental factors such as stress and trauma. As a result, epigenetic alterations may serve as a biological record of abuse, offering crucial insights into the mechanisms linking early adverse experiences to long-term mental health outcomes and the risk of developing chronic diseases [74].

Table 2 summarizes the translational studies employing three main epigenetic targets investigated in abused survivors:

• DNA methylation: increased methylation of the *NR3C1* gene (glucocorticoid receptor) is linked to an exaggerated stress response [75,76,77].
• Histone modifications: alterations in histone acetylation impact genes regulating emotional and behavioral responses [78,79].
• MicroRNA (miRNA) dysregulation: miRNAs play a role in neuroinflammation and synaptic plasticity, potentially contributing to psychiatric disorders in abuse survivors [80,81,82].

By delving into these molecular alterations, researchers aim to gain a deeper understanding of how child abuse imprints on biological systems, potentially paving the way for more targeted interventions and support mechanisms for affected individuals [83].

## 6. Current Interventions and Treatment Strategies for Child Abuse

A comprehensive, multidisciplinary evaluation is essential, integrating forensic medicine, psychology, and genetics to ensure accurate diagnosis and appropriate intervention based on the child’s need. Current interventions and treatment strategies for child abuse focus on addressing the immediate safety and psychological needs of the child, as well as preventing further abuse. To effectively address this challenge, we must encourage and explore more advanced methodologies that not only provide early intervention and care within the narrowest possible timeframe to mitigate developmental compromises, but also prioritize the systematic collection and sharing of data, ongoing case monitoring, and the comprehensive documentation of the clinical and socio-familial progression of maltreatment. Treatment and therapeutic approaches should be diversified and intensified, integrating clinical outcomes with molecular investigations. The development of personalized assessment tools, such as genomic and epigenetic panels, represents a concrete opportunity to analyze the phenotypic manifestations of maltreatment and to further elucidate the cellular and genomic responses underlying neuroendocrine mechanisms. The establishment of multidisciplinary teams would maximize collaborative efforts, enhance the focus on medical perspectives, and resolve many remaining forensic challenges.

A shift towards multidisciplinary collaboration has the potential to significantly disrupt the cycle of violence and the intergenerational transmission of maladaptive psychobehavioral patterns. A particularly compelling aspect of an integrated system could involve the monitoring of pregnant women; for example, fetal skeletal development analysis can refine indicators of skeletal stress related to prenatal experiences and, implicitly, assist in monitoring maternal stress and safeguarding women who may be experiencing current or past violence [84]. Emergency interventions prioritize creating a safe environment for the child, which may involve removing them from an abusive setting [85,86,87]. This is often followed by a thorough assessment to understand the extent of physical and psychological harm. Psychological interventions are critical in addressing the trauma associated with abuse. Trauma-focused cognitive behavioral therapy is one of the most effective treatments, helping children process their experiences, develop coping mechanisms, and reduce symptoms of anxiety and PTSD/CAS. In addition, support for non-abusive caregivers is essential to provide a stable and safe environment for the child [12,88].

Family therapy can facilitate communication, strengthen relationships, and promote healing within the family unit. On a broader level, public health strategies emphasize the importance of prevention programs that educate communities, identify risk factors, and promote protective measures to prevent abuse from occurring. Training for professionals in recognizing and responding to signs of abuse is vital to ensuring early intervention and support [1,12]. Furthermore, participants who have a higher level of self-perceived competence are significantly more willing to identify and manage cases, although this self-assessment has not been shown to correlate with their actual skills or level of willingness to intervene effectively. This study concludes that there is a pressing need for specialized training programs tailored to enhance the skill sets of healthcare providers in identifying and managing child abuse and neglect. These programs should encompass not only academic knowledge but also practical applications and psychosocial support techniques to ensure a holistic approach to combating this serious issue. In terms of child protection training for professionals to improve reporting of child abuse and neglect, there is a lack of strong evidence. It is unclear whether child protection training is better than no training or alternative training (e.g., cultural sensitivity training) at improving professionals’ reporting of child abuse and neglect. Larger, well-designed studies are needed to assess the effects of training with a wider range of professional groups. Future research should compare face-to-face with e-learning interventions to improve the reporting of child abuse and neglect. Many professional groups, such as teachers, nurses, doctors, and the police, are required by law or organizational policy to report known or suspected cases of child abuse and neglect to statutory child protection authorities. To prepare them for reporting, various training interventions have been developed and used. These can vary in duration, format, and delivery methods. For example, they may aim to increase knowledge and awareness of the indicators of child abuse and neglect; the nature of reporting duty and procedures; and attitudes towards reporting duty. Such training is usually undertaken post-qualification as a form of continuing professional development; however, little is known about whether training works, either in improving reporting of child abuse and neglect generally, for different types of professionals, or for different types of abuse.

By integrating these strategies, current interventions aim to not only treat the consequences of abuse but also to prevent it and promote long-term healing and resilience in affected children [89]. In line with a modern approach of ELS risk factor identification [90], the most effective approach involves prioritizing prevention through increased awareness and education for both children and healthcare providers. The current lack of a coordinated and efficient protocol for diagnosing child abuse and maltreatment, and for managing the long-term consequences, necessitates targeted interventions to protect potential victims and minimize the risk of abuse. Identifying and supporting at-risk children is crucial for early intervention and improved outcomes (Table 3).

Recent evidence highlights that certain groups of children are disproportionately vulnerable to the effects of abuse, not due to inherent characteristics, but because of overlapping social, psychological, and environmental stressors. While this review avoids deterministic labeling, it is crucial to recognize and understand the contextual risk factors that increase exposure and reduce resilience to maltreatment. Female children and young women often face higher rates of sexual and gender-based violence [91,92], while children with special needs may be at increased risk due to communication barriers, dependency, or lack of appropriate care structures [93]. A history of intra-familial abuse or domestic violence significantly raises the likelihood of continued exposure to harm [94]. Similarly, children in institutional or foster care settings often experience disruptions in stable adult relationships, compounding their vulnerability [95]. Children facing bereavement may suffer emotional instability that can impair their ability to seek help or be recognized as in distress [96,97]. LGBTQ+ youth are frequently subjected to discrimination, rejection, and identity-based victimization, which can increase their risk of abuse both at home and in peer settings [98]. Children exposed to bullying or peer-on-peer abuse, particularly in school environments, often show psychological distress that can be compounded by a lack of institutional support [99,100]. It is important to note that some of these experiences—such as child-on-child sexual assault—may not always be captured under traditional definitions of child sexual abuse, yet they can have similarly profound and lasting psychological impacts.

Black and Minority Ethnic (BME) children [101] and Unaccompanied Foreign Minors (UFMs) [102,103] may face systemic inequities, language and cultural barriers, and limited access to services, which can all hinder detection and intervention in abuse cases. Recognizing these groups as vulnerable is not intended to stigmatize, but rather to guide the development of protective, inclusive, and proactive frameworks that ensure early intervention. By shifting the focus from reactive measures to anticipatory care and tailored support systems, healthcare professionals and policymakers can work towards minimizing risk and fostering resilience among children most at risk of being overlooked or underserved.

Future research in the field of child abuse correlates is poised to delve deeper into the integration of clinical and molecular data to better understand and address the long-term impacts on victims. One promising direction is the exploration of genetic and epigenetic markers that may predispose individuals to different responses to abuse, including resilience or susceptibility to mental health issues. Advances in technology, particularly in genomics and proteomics, are enabling researchers to identify specific biomarkers associated with trauma, which can lead to more personalized therapeutic interventions [50,89]. Moreover, the intersection of digital health tools and big data offers unprecedented opportunities to gather large-scale, longitudinal data sets that can illuminate patterns and correlations previously undetectable. The integration of artificial intelligence and machine learning in analyzing these complex data sets could lead to the development of predictive models that help identify at-risk children earlier and provide insights into effective prevention strategies [104].

It is also vital to focus on translational research that bridges laboratory findings with clinical practice. There is a growing need to design interventions that are informed by molecular research, which could revolutionize treatment modalities for survivors of child abuse. Furthermore, interdisciplinary collaborations between clinicians, molecular biologists, psychologists, and social workers will be essential in translating research findings into policy and practice, ultimately aiming to reduce the incidence and impact of child abuse globally [1,85,86,87]. Advances in understanding molecular mechanisms are helping to translate into clinical practice, offering the potential to bolster individual resilience to early life stress and design personalized interventions for children and adults who have experienced child abuse and maltreatment. Exciting avenues for this include pharmacogenomics [105,106,107], which tailors medications based on genetic predispositions to stress responses; epigenetic therapies, such as exploring histone deacetylase inhibitors for PTSD treatment [108,109]; and digital health tools, utilizing AI-driven monitoring systems for real-time assessment and intervention [110].

Several factors may mask, mitigate, or prevent the manifestation of structural chromosome aberrations due to intrinsic and extrinsic characteristics (e.g., number, type, duration, reiteration, age, controllability, family-related/unrelated offender) and the stressor itself [111]. Stress involves constant interactions with the surrounding environment, promoting various types of emotional and behavioral responses, which in turn further shape the interaction with the stressor. There is still a lack of comprehensive analysis on the measurable effects of single versus multiple stressors, including factors such as the timing within specific developmental windows, the duration and repetition of stress, the victim’s age, the nature of the offender, the presence of socio-environmental protective interventions, stress reliver practices and individual psychological responses. Effective management of the pathophysiological consequences of ELS could be achieved through the implementation of therapeutic protocols that integrate mindfulness-based techniques with socio-affective and socio-cognitive practices, and especially with appropriate support from healthcare providers, avoiding additional distress for the victims [112]. These interventions have been shown to reduce perceived stress not only in victims, but also in their caregivers. Such a strategy would systematize the response to ELS by enhancing anti-inflammatory pathways and cellular protective mechanisms, while also allowing for qualitative and quantitative assessment of health progression.

## 7. Conclusions

Child abuse is a multifaceted issue with profound clinical and molecular repercussions. Translational research integrating genetics, epigenetics, and neurobiology with clinical practice holds promise for improving detection, intervention, and long-term outcomes for survivors. Future research should prioritize interdisciplinary collaborations to develop personalized, evidence-based approaches to mitigating the impact of abuse.

## Figures and Tables

**Table 1:** Studies utilizing translational approaches in child abuse research to examine the genetic susceptibility of ELS-related disorders. *PTSD (posttraumatic stress disorder); MDD (major depression disorder)*.

Gene	Risk Allelic Variants	Clinical Implications	Ref.
*FKBP5*	rs1360780 and rs3800373	PTSD	[68]
*BDNF*	rs6265	depression	[69]
*SLC6A4*	5-HTTLPR-s allele	MDD	[70]
*MAOA*	MAOA-L allele	antisocial behavior	[71]
*CRHR1*	rs7209436, rs4792887, rs110402, rs242924, and rs242939	depression/suicidal behavior	[72]

**Table 2:** Notable studies utilizing translational approaches in child abuse research.

Epigenetic Target	Key Findings	Clinical Implications	Ref.
DNA methylation (*NR3C1*)	Elevated methylation in abuse survivors	Potential target for stress-response modulation	[75,76,77]
Histone modifications	Increased inflammatory response in maltreated children	Biomarker-based psychiatric risk assessment	[78,79]
miRNA profiles	miRNA alterations linked to PTSD and depression	Epigenetic therapy prospects	[80,81,82]

**Table 3:** Risk profiles for child abuse and maltreatment.

Risk Group	Description	Ref.
Female Children & Young Women	Girls and young women are disproportionately affected	[91,92]
Children with Special Needs	Children with special educational needs and disabilities	[93]
History of Intra-Familial Abuse or Domestic Violence	Children with prior experience of abuse within the family or living in a home with domestic violence	[94]
Children in Care	Children in the care of social services or other institutions	[95]
Children Experiencing Bereavement	Children who have experienced the loss of a parent, sibling, or friend	[96,97]
LGBTQ+ Children	Children who are perceived as, or identify as, LGBTQ+	[98]
Children Experiencing Bullying/Child-on-Child Abuse	Children who are victims of bullying or involved in child-on-child abuse	[99,100]
Black and Minority Ethnic (BME) Children	Children from BME backgrounds who may be under identified as victims or over-identified as perpetrators	[101]
Unaccompanied Foreign Minors (UFMs)	Children who have entered a country without a parent or legal guardian	[102,103]

## Data Availability

Not applicable.

## Supplementary Materials

The following supporting information can be downloaded at: https://www.mdpi.com/article/10.3390/children12060797/s1, Figure S1: Schematic illustration of the PRISMA flowchart used in the present review.
