# Supplementary material for: The Many Faces of Child Abuse: How Clinical, Genetic and Epigenetic Correlates Help Us See the Full Picture
Source: Children (Basel). 2025 Jun 18;12(6):797. doi: 10.3390/children12060797 (PMC12191296; doi:10.3390/children12060797)

Supplementary Figure S1. Schematic illustration of the PRISMA flowchart used in the present review.

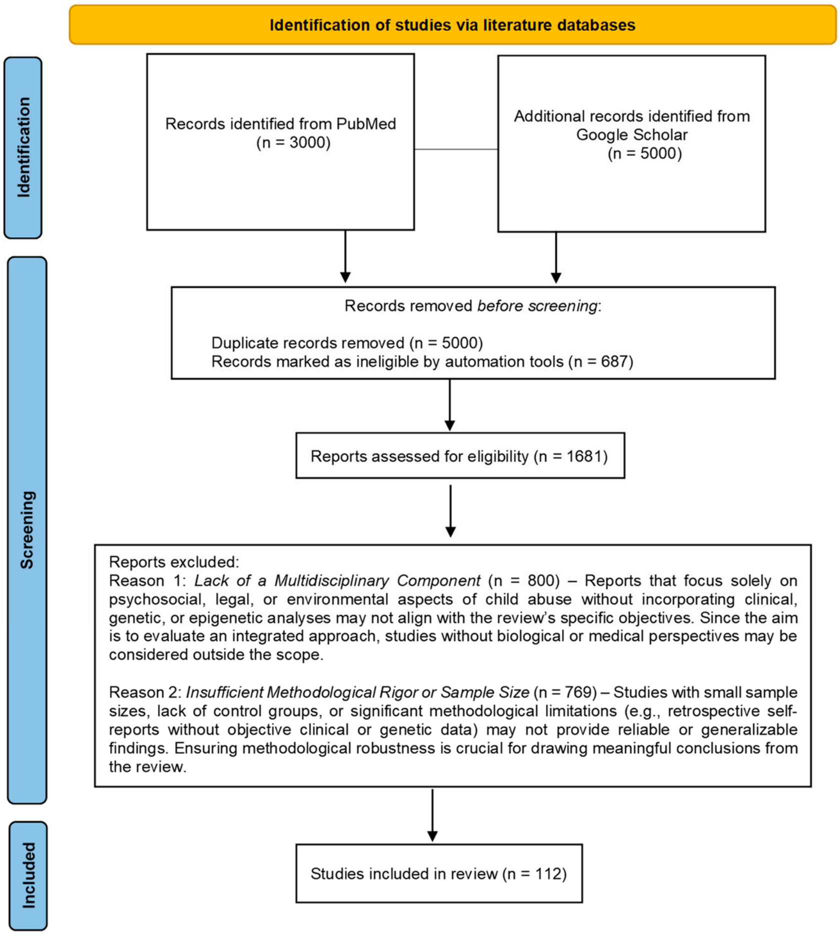

Supplement: Supplementary file 1 [file children-12-00797-s001.zip › children-3658892-supplementary.pdf]
